# Protective effects of *Forsythiae fructus* and *Cassiae semen* water extract against memory deficits through the gut-microbiome-brain axis in an Alzheimer’s disease model

**DOI:** 10.1080/13880209.2022.2025860

**Published:** 2022-01-25

**Authors:** Da Sol Kim, Ting Zhang, Sunmin Park

**Affiliations:** Food & Nutrition, Obesity/Diabetes Center, Hoseo University, Asan, South Korea

**Keywords:** Amyloid-β, neuroinflammation, hippocampal insulin signalling, memory function, gut-brain axis

## Abstract

**Context:**

Fruits of *Forsythia suspensa* Vahl (Oleaceae) and seeds of *Cassia obtusifolia* Linne (Caesalpinaceae) have been used to treat inflammation in Asia.

**Objective:**

We examined the alleviation of memory function in Alzheimer's disease (AD) rats fed *Forsythiae Fructus* (FF) and *Cassiae Semen* water extracts (CS) and investigated the mechanisms responsible for the effects.

**Materials and methods:**

Thirty Sprague-Dawley male rats had hippocampal infusions of amyloid-β_(25–35)_ (AD rats; memory deficit), and ten rats were infused with amyloid-β_(35–25)_ (non-AD rats; no memory deficit). For eight weeks, all rats freely consumed high-fat diets (43% lard) incorporated with 200 mg/kg body weight assigned aqueous herbal extracts: AD-FF, AD-CS, or without extracts AD-CON (control), non-AD (normal-control).

**Results:**

Memory impairment was prevented in the AD-FF (0.54 ± 0.06-fold) and the AD-CS rats (0.33 ± 0.04-fold) compared to the AD-CON by inhibiting amyloid-β deposition to the levels less than one-fourth of the AD-CON group. The hippocampal pAkt→pGSK-3β→pFOXO1 pathway was attenuated by approximately 3.25-fold in the AD-CON, while AD-FF prevented the attenuation better than AD-CS. The relative intensity of hippocampal tau protein based on β-actin was suppressed with AD-FF (0.68 ± 0.09) and AD-CS (0.96 ± 0.81), compared to AD-CON (1.19 ± 0.13). AD decreased the abundance of *Bacteroidales* by 34.2% and *Lactobacillales* by 23.8% and increased *Clostridiales* by 181% while the AD-FF, but not the AD-CS, normalised the gut microbiota changes to be similar to the non-AD.

**Discussion and conclusions:**

FF improved memory deficits better than CS in an AD-induced rat model. The potential neuroprotective benefits of FF against AD may be applicable to human AD therapy with additional clinical research.

## Introduction

Dementia is associated with brain cell death caused by cerebral ischaemia or Alzheimer’s disease (AD) (Kim et al. 2011; Park et al. 2013). Dementia from cerebral ischaemia occurs in middle-aged adults, and treatment targets include dyslipidemia and platelet aggregation to prevent ischaemia-related dementia (Kim et al. 2011). However, AD is a slowly progressive neurodegenerative disease characterised by amyloid-β accumulation and reductions in cholinergic neuron numbers caused by hippocampal cell death (Skovgard et al. 2018), eventually resulting in loss of cognitive function, disability, and dementia. The prevalence of AD is age-dependent, and the disease is common in those over age 70 (Hodson 2018). Most patients with dementia (75%) have AD, and although the cause of AD remains unknown, it is known to be associated with neuronal cell death caused by amyloid-β accumulation in the brain, including the hippocampus (Hodson 2018).

Therapeutic targets include decreasing tau phosphorylation by potentiating hippocampal insulin signalling and increasing neurotrophic factors, including brain-derived neurotrophic factor (BDNF), which is involved in neurogenesis (Amidfar et al. 2020). AD is also associated with acetylcholine concentrations and neuroinflammation in the hippocampus (Ferreira-Vieira et al. 2016; Park et al. 2017a). Since the aetiology of AD is multifactorial, drugs limited to one target may not be sufficient to confer protection (Hodson 2018). Herbs contain various components that might prevent and delay disease progression by modulating hippocampal insulin signalling and potentiating insulin sensitivity, especially in the hippocampus, thus preventing and alleviating the progression and symptoms of AD (Chen et al. 2020). Accumulating evidence suggests an association of gut microbiota with Alzheimer’s disease through the gut-microbiota-brain axis, especially gut bacteria that produce short-chain fatty acids (SCFA) and modulate inflammation, immune response endocrine regulation, including enteric hormones, and hypothalamic-pituitary-adrenal (HPA) axis, and neurotransmitter regulation. Consumption of probiotics, prebiotics, and herbal extracts modulates pro-inflammatory cytokines, amino acids, and hormone metabolisms to alleviate neuroinflammation and amyloid-β accumulation and protect against Alzheimer’s disease.

*Forsythiae Fructus* is the dry fruit of *Forsythia suspensa* Vahl (Oleaceae), and *Cassiae* Semen is the seed of *Casssia obtusifolia* Linne (Caesalpinaceae). They have been used as herbal medicines, as reported in Donguibogam in Asian countries (Heo 1610). In a preliminary study, water extracts of *Forsythiae Fructus* (FF) and *Cassiae Semen* (CS) prevented neuronal cell death by amyloid-β_(25–35)_ when we tested the effects of about 50 herbal extracts on amyloid-β_(25–35)_ induced cell death using PC12 cells differentiated with neuronal growth factor (NGF). FF contains lignans, lignan glycosides, and flavonoids, whereas its major components are phylligenin, betulinic acid, oleanolic acid-3-acetate, arctiin, isorengyol, and matairesinoside (Lee et al. 2010). The effects of FF on the prevention of neuronal cell death have not been studied in Alzheimer’s disease-induced animals. FF, including phylligenin, reduced the productions of reactive oxygen species (ROS) and nitric oxide (NO) in rat glial cells *in vitro* (Lee et al. 2010). On the other hand, CS contains anthraquinones such as chrysophanol, emodin, physcion, obtusifolin, aloe-emodin, alaternin, and aurantio-obtusin (Guo et al. 2017). Its extracts have been reported to exhibit antihyperlipidemic, antidiabetic, neuroprotective, hepatoprotective, and hypotensive activities (Dong et al. 2017a). Protection against AD development and progression by CS is associated with its antibacterial, antioxidant, and anti-inflammatory activities (Dong et al. 2017a). Furthermore, its anti-inflammatory activity may be linked to modulation of the gut microbiome since herbal components impact bacterial growth and change gut bacterial compositions in a manner that reduces inflammation (An et al. 2019). Moreover, gut microbiome byproducts, such as short-chain fatty acids (SCFA), cytokines, immune cells, amines, and neuropeptides, play significant roles in the gut-brain axis (Quigley 2017; Wei et al. 2020).

We hypothesised that supplementation with FF or CS might improve memory function by reducing amyloid-β accumulation in the hippocampus and potentiating insulin/insulin-like growth factor-1 signalling. The study was performed using rats infused with amyloid-β_(25–35)_ into the hippocampus. This method induces memory loss due to hippocampal amyloid-β accumulation in rodent models (Schimidt et al. 2017; Park et al. 2017a).

## Materials and methods

### Extraction, lyophilisation, and quantification of phenolics

*Forsythiae Fructus* and *Cassiae Semen* were purchased from an herbal market on March 20, 2019, and their identities were confirmed by Dr. Young Seung Joo **(**Woosuk University, Jeonju, Korea). *Forsythiae Fructus* and *Cassiae Semen* were stored as voucher specimens HU-H84 and HU-H89, respectively. Both were extracted by immersion in hot water (4 h at 95 °C) and then centrifuged at 8,000 × *g* for 30 min. Supernatants were collected and lyophilised in a freeze-drier (Il Shin, Dongdochun-Si, Korea). The yields of FF and CS were 17.5 and 13.3%, respectively. As previously described, concentrations of total phenolics were determined using the Folin–Ciocalteu method (Ko et al. 2014). After 3 min, 10% (w/v) Na_2_CO_3_ was added to each reaction mixture of each herbal extract. Reactions were performed in the dark for 60 min, and absorbances at 725 nm were recorded using a UV spectrophotometer (JASCO, Japan). Total flavonoid contents were measured using the methods designed by Davis (AOAC 2012), with some modifications. Each extract was mixed with 5% sodium nitrite, and after 5 min, 10% aluminium chloride (3:1:1, v/v/v; Sigma) was added. Each mixture was neutralised with 1 N NaOH after incubating it at room temperature for 6 min, and its optical density was measured at 510 nm using a UV spectrophotometer (Perkin Elmer). The total phenol contents were expressed as mg gallic acid equivalents per gram.

### Animal care, surgical procedures, and diet preparation

Forty male Sprague-Dawley rats aged eight weeks were purchased from Daehan Bio Inc. (Eum-Sung, Korea) and acclimatised for seven days in an animal facility. Each animal was housed in an individual stainless-steel cage in a controlled environment (23 °C, 12 h light/dark cycle) with *ad libitum* access to food and water. All procedures conformed with the Guide for the Care and Use of Laboratory Animals (8^th^ edition) issued by the National Institutes of Health and were approved by the Institutional Animal Care and Use Committee of Hoseo University (HSIACUC-17-071).

**Figure 1.**
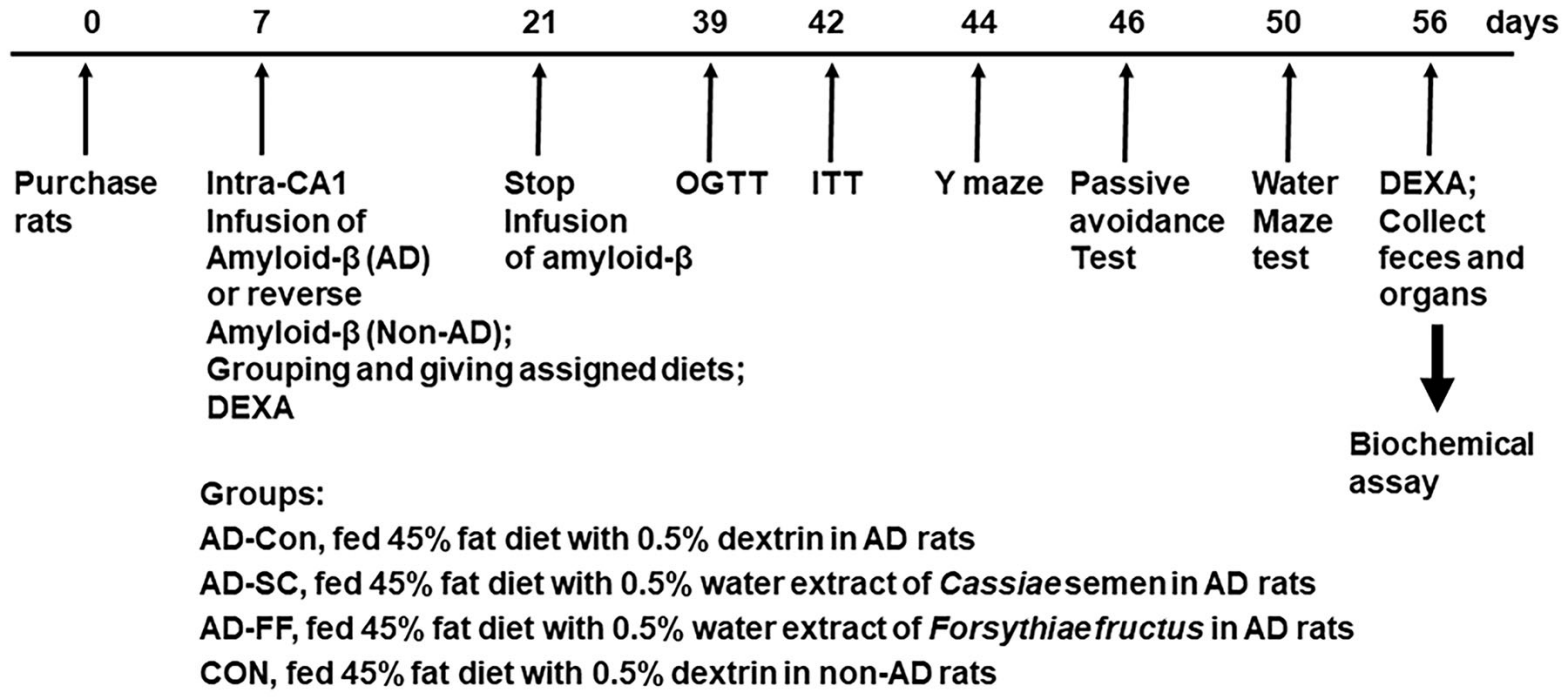
Experimental design.

After the 7-day acclimation period, rats weighed 209 ± 14 g. Animals were anaesthetised with an intraperitoneal injection of ketamine and xylazine (100 and 10 mg/kg body weight, respectively) and placed in a stereotaxic device. Two catheters were bilaterally placed in the CA1 region of the hippocampus with the coordinates of 4.5 mm anterior to bregma, 1.6 mm ventral to the dura, and two directions of ± 4.0 mm from the interaural line (Park et al. 2013; Yang et al. 2013). Amyloid-β_(25–35)_ or _(35–25)_ was dissolved in sterile saline and infused into the CA1 region using an osmotic pump (Alzet Osmotic Pump Company; Cupertino, CA, USA) at a rate of 3.6 nmol/day for 14 days. The cannula was connected to 22-gauge tubing filled with amyloid-β_(25–35)_ or amyloid-β_(35–25)_. Amyloid-β_(35–25)_ was used as a normal-control sequence instead of saline since amyloid-β_(25–35)_ may have adverse effects other than amyloid-β accumulation. Amyloid-β_(25–35)_ can aggregate and form deposits in the hippocampus leading to the development of Alzheimer’s diseases (AD rats), but amyloid-β_(35–25)_, a reversed amyloid-β_(25–35)_ sequence, does not aggregate in the brain (non-AD rats). Assigned diets were provided from the catheter insertion (day 1) until sacrifice on day 49. A schematic of the experimental procedure is given in Figure 1.

We estimated the FF and CS dosages based on the dosage to prevent cell death by amyloid-β_(25–35)_ in the PC12 cell line (a pheochromocytoma cell line). Treatments with CS or FF water extracts (5–20 μg/mL) were found to reduce amyloid-β_(25–35)_-induced cell death as determined by 3-(4,5-dimethylthiazol-2-yl)-2,5-diphenyltetrazolium bromide (MTT) assay. Based on the preliminary cell culture results, their utilisation rates (20%) and half-life (about 4 h), animal dosages were calculated and were about 200 mg/kg body weight/day (Committee of FDA 2005; Manach et al. 2005).

The 40 rats were allocated to four groups of 10 rats; test ingredients were incorporated into a high-fat diet (HFD) made with semi-purified AIN-93 formulation (Park et al. 2018b). The four treatment groups were as follows: AD rats that received dextrin as control (the AD-CON group), CS (200 mg/kg body weight; the AD-CS group), or FF (200 mg/kg body weight; the AD-FF group). Non-AD rats were administered dextrin as normal control (the CON group). To reduce the stress caused by oral administration of FF and CS water extracts by gavage twice a day, FF or CS were added to the diet at 0.5% (w/w). High-fat diets in the AD-CON and the CON groups contained 0.5% indigestible dextrin (Cuwon, Seoul, Korea) instead of CS or FF. All rats had free access to a high-fat diet made by a modified polyphenol-free AIN-93 semi-purified method, which has been shown to induce insulin resistance and exacerbate amyloid-β accumulation in AD rats (Park et al. 2013). The symptomatic differences of AD-CON and CON groups were related only to amyloid-β infusion since the animals of the AD-CON and CON groups were also fed a high-fat diet. CS or FF water extracts were homogeneously blended with a vitamin/mineral mixture and sugar, sieved, and mixed with feeds containing 37% carbohydrate (cornstarch and sucrose), 20% protein (casein), and 43% lard (CJ Co., Seoul). Feeds were then re-sieved and stored at 4 °C. Each diet had equivalent energy (4.65 kcal/g) and nutrient composition. All rats, including the AD-CON and CON groups, consumed a high-fat diet to determine the CS and FF effect on AD. All rats had free access to water and experimental diets during the entire experimental period. A new diet was provided every other day, and food intakes were measured every week, and CS and FF intakes were calculated from their food intake.

### Body composition and oral glucose tolerance test (OGTT)

Body compositions were determined by dual-energy X-ray absorptiometry (DEXA), and the units were calibrated using a phantom supplied by the manufacturer (Norland Medical Systems Inc., Fort Atkinson, WI, USA) before and after providing assigned diets. After anaesthetising rats with the ketamine/xylazine mixture (100 and 10 mg/kg body weight, respectively), animals were placed prone with rear legs held in external rotation with tape. After scanning, bone mineral density (BMD) in the lumbar spine, lean body masses (LBM) in the hip and leg, and fat masses in the abdomen were calculated using the software for rodents supplied with the DEXA instrument (Park et al. 2017b).

Oral glucose administration (2 g/kg body weight) was conducted after an overnight fast, and serum glucose concentrations were measured every 10 min for 90 min and again at 120 min using a Glucometer (Accuchek, Roche Diagnostics; Basel, Switzerland). Two days later, insulin was injected intraperitoneally after 6 h of food deprivation, and serum glucose concentrations were measured for intraperitoneal insulin tolerance testing (IPITT). Serum insulin concentrations in the fasting state were determined using an ultrasensitive enzyme-linked immunosorbent assay (ELISA) kit (Crystal Chem, Elk Grove Village, IL, USA). Homeostasis model assessment for insulin resistance indices (HOMA-IR) was calculated as follows: serum insulin (μU) × serum glucose (mmol/L)/22.5. Serum TNF-α levels were measured using an ELISA kit (eBioscience; San Diego, CA, USA).

### Memory deficit assessments using the passive avoidance and Y and water maze tests

Memory deficits were assessed using a passive avoidance apparatus equipped with a two-compartment dark/light shuttle box (Yang et al. 2013). Electroshocks (75 V, 0.2 mA, 50 Hz) was delivered for 5 s when a rat entered the dark chamber in the acquisition trial. The rat was moved from the dark chamber to its home cage 5 s later. After 24 h, the time taken to enter the dark chamber (latency) was reassessed in the same manner, but no electric foot shock was delivered. Latency was measured up to a maximum of 600 s.

The Y maze test consisted of a horizontal Y-shaped maze with three arms of 50.5 cm in length, 20 cm in width, and 20 cm in height. A rat was placed in one arm, and its movements were monitored for 8 min. A correct alternation indicated consecutive entry into all three arms of the Y maze (Ko et al. 2019). The percentage of spontaneous alternations was calculated by expressing the correct alternations as a percentage of total arm entries.

As previously described, spatial memory function was assessed using the Morris water maze test (Park et al. 2013; Yang et al. 2013). This test provides assessments of hippocampal-dependent learning, including the acquisition of spatial memory. Swims were started in zone 1 of the pool for days 1 and 3 trials where the platform was located in zone 5. On day 5, the platform was removed, and latency time to swim to and stay in zone 5 was measured. The test was conducted with a cut-off time of 600 s. Memory deficiencies were assessed using times spent in zone 5 and times taken to reach zone 5.

### Tissue collection and assays at the end of the intervention

Two days after the water maze test, all rats were deprived of food for 16 h and then anaesthetised with a mixture of ketamine and xylazine. Human regular insulin (5 U/kg body weight) was then injected through the inferior vena cava of the rats. Ten min later, the rats were killed by decapitation, and blood from the portal vein, the inferior vena cava, and organs were rapidly collected. The epididymal and retroperitoneal fat masses were weighed. Six brains randomly selected from each group were frozen at −70 °C, while the rest (*n* = 4) were fixed in the 4% paraformaldehyde. The faeces in the caecum were collected and frozen at −70 °C.

The hippocampi of six rats were divided into two sections: three out of 6 were lysed with RIPA buffer containing protease inhibitors, and the remaining hippocampi were randomly selected to be lysed with Trizol for extracting total RNA. The hippocampal RIPA buffer lysates were centrifuged at 5,000 *g* for 10 min, and their supernatants were used to measure lipid peroxide and triglyceride contents by colorimetry using a Lipid peroxidation (MDA) assay kit (Abcam, Cambridge, UK). The supernatants were deproteinized with 1.5 N perchloric acid, and the glycogen content was calculated from glucose concentrations derived from glycogen hydrolysed by α-amyloglucosidase in an acid buffer (Frontoni et al. 1991). Glucose concentrations were measured with a glucose kit (Asan Pharmaceutics, Seoul, Korea). Triglyceride was extracted with a chloroform-methanol (2:1, v/v) from the hippocampus and resuspended in pure chloroform (Kwon et al. 2013), and triglyceride in the chloroform was measured by TG kit (Asan Pharmaceutics, Seoul, Korea). Serum aspartate aminotransferase (AST) and alanine aminotransferase (ALT) concentrations were measured using Colorimetry AST and ALT kits (Asan Pharmaceutics, Seoul, Korea).

### Quantitative realtime PCR, immunohistochemistry, and immunoblotting

The hippocampal tissues were collected from three randomly selected rats per group. Total RNA was isolated from tissues using a monophasic solution of phenol and guanidine isothiocyanate (Trizol reagent, Invitrogen, Rockville, MD, USA). cDNA was produced using a mixture of total RNA, superscript III reverse transcriptase, and high fidelity Taq DNA polymerase (1:1:1, v:v:v) by polymerase chain reaction (PCR) and mixed with the primers of genes of interest and SYBR Green mix. The expressions of genes of interest were determined using a realtime PCR machine (BioRad Laboratories, Hercules, CA, USA). The primers used for ciliary neurotrophic factor (CNTF), BDNF, TNF-α, IL-1β, and β-actin were as previously described (Peinnequin et al. 2004). Gene expression levels in samples were quantitated using the comparative cycle of threshold (CT) method (Livak and Schmittgen 2001).

The brains of four rats per group were perfused *in situ* with saline and 4% paraformaldehyde solution (pH 7.2) and postfixed using the same fixative overnight at room temperature (Park et al. 2018b). The fixed brain was put into a 30% sucrose solution overnight and cryoprotected. Frozen tissues were serially sectioned coronally using a cryostat (Leica, Wetzlar, Germany) at 30 μm in six-well plates containing phosphate-buffered saline. Hippocampi were immunostained with an anti-amyloid-β antibody, and amyloid deposits were stained with fluorescent secondary antibodies.

Hippocampal tissues from four rats from each group were dissected as previously described. Each tissue was lysed with RIPA lysis buffer containing protease inhibitors, and the lysate protein contents were measured using a Bio-Rad protein assay kit (Hercules, CA, USA). Each lysate from two rats was used for immunoblotting that was performed in two sets. The lysates having protein (30–50 μg) were resolved into sodium dodecyl sulfate-polyacrylamide gel electrophoresis, and the amount of the interest proteins was examined with the specific antibodies as follows: protein kinase B (PKB or Akt), phosphorylated PKB^Ser473^, glycogen synthase kinase (GSK)-3β, phosphorylated GSK-3β^ser9^, forkhead Box O1 (FOXO1), phosphorylated FOXO1^Ser249^, phosphorylated tau^ser396^ and tau (Cell Signalling Technology, Danvers, MA, USA) and β-actin (Santa Cruz Biotech, Dallas, TX, USA). The intensity of the interest proteins was measured using Imagequant TL (Amersham Biosciences, Piscataway, NJ, USA).

### Serum SCFA concentrations and gut microbiome by next-generation sequencing (NGS)

Serum separated from the portal vein blood was mixed with ethanol (Duksan, Korea), and 1 N HCl (100:1) was added to the mixture. It was vortexed and centrifuged at 15 000 rpm, 15 min, and 4 °C. SCFA concentrations in supernatants were measured by gas chromatography (GC, Clarus 680 GAS, PerkinElmer) using an Elite-FFAP 30 m × 0.25 mm × 0.25 μm capillary column with helium as the carrier gas at a flow rate of 1 mL/min, as described previously (Park et al. 2021) Exogenous acetate, propionate, and butyrate (Sigma Co., MO, USA) were used for references.

Faecal microbiome communities were investigated using faeces from caeca by metagenome sequencing using next-generation sequencing procedures (Park et al. 2018a). Bacterial DNA was extracted from faeces using a Power Water DNA Isolation Kit (Qiagen, Valencia, CA, USA), according to the manufacturer’s instructions. DNA was amplified with 16S amplicon primers by PCR, and libraries were prepared for PCR products according to the GS FLX plus library prep guide, as described previously (Wu et al. 2021). According to the manufacturer’s instructions, the PCR amplification program was run with 16S universal primers in the FastStart High Fidelity PCR System (Roche, Basel, Switzerland). Sequencing of bacterial DNA in faeces was performed using the Illumina MiSeq standard operating procedure and a Genome Sequencer FLX plus (454 Life Sciences) (Macrogen, Seoul).

16S amplicon sequences were processed using Mothur v.1.36. Miseq SOP was used to identify faecal bacterial taxonomy, and bacterial counts were conducted on each faecal sample. Sequences were aligned using Silva reference alignment v.12350, and bacteria counts and identifications for all taxa were determined as previously described (Park et al. 2021; Wu et al. 2021). Relative bacteria counts were calculated in taxonomic assignment order for each sample. PCoA results for gut bacteria were visualised using the R package.

### Statistical analyses

The statistical analysis was performed using SAS version 7 (SAS Institute; Cary, NC, USA). A sample size of 10 per group was determined using the G power program (power = 0.85 and effect size = 0.50) to test the main effects. Results are expressed as means ± standard deviations (SDs). Univariate analysis was used to analyse normally distributed variables. One-way ANOVA was used to compare groups, and multiple comparisons were conducted with the Tukey test when one-way ANOVA showed a significant intergroup difference. Statistical significance was accepted for *p*-values < 0.05.

## Results

### Total phenol and flavonoid contents in the CS and FF

CS and FF extractions yielded 312 ± 1.2 and 328 ± 1.6 mg/g dried extract, and total polyphenolic contents were 67.0 ± 0.8 in CS extract and 45.1 ± 0.7 mg/g powder in FF extract (Table 1).

**Table 1.** Contents of polyphenols and flavonoids in water extract of *Cassiae* Semen (CS) and *Forsythiae Fructus* (FF).

	CS	FF
Total polyphenols (mg GAE/g dried extract)	312 ± 1.2	328 ± 1.6
Total flavonoids (mg QE/g dried extract)	67 ± 0.8	45.1 ± 0.7

Values represent means ± standard deviations (*n* = 3).

### Energy metabolism, body composition, and liver damage

Seven weeks after initiating amyloid-β infusion, body weights and weight gains were not significantly different between the AD-CON and CON groups but were higher in the AD-CS group than in other groups (*p* < 0.05; Table 2). Epididymal fat and retroperitoneal fat masses were not significantly different among the four groups. Visceral fat mass was not different between AD-CON and CON but was lowered in AD-FF (*p* < 0.05; Table 2). Bodyweight gain was related to food intake in the AD-CS group, and these rats had higher food intakes than those in the AD-CON group. Food intake tended to be higher in the CON group than in the AD-CON group (*p* < 0.05; Table 1).

**Table 2.** Body weight and glucose metabolism at the end of experimental periods.

Variables	AD-Con	AD-CS	AD-FF	CON
Body weight (g)	401 ± 6.5	425 ± 10*	396 ± 9.5	400 ± 10.3*
Weight gain for 5 weeks (g)	191 ± 7.6	216 ± 12*	187 ± 6.9	191 ± 9.8*
Epididymal fat pads (g/kg bw)	4.9 ± 0.4	4.9 ± 0.8	4.3 ± 0.3	4.6 ± 0.5
Retroperitoneal fat (g/kg bw)	5.8 ± 0.6	5.7 ± 0.7	5.2 ± 0.6	5.3 ± 0.6
Visceral fat mass (g/kg bw)	10.7 ± 1.1	10.6 ± 1.5	9.5 ± 8.4*	9.9 ± 10.1
Food intake (g)	15.2 ± 1.1	17.3 ± 1.2*	15.9 ± 0.8	16.4 ± 1.2
CS or FF intake (mg/kg bw/day)	–	204 ± 14.2	200 ± 11	–
Fasting serum glucose (mg/dL)	102 ± 7.4	101 ± 5.0	98.8 ± 4.6	99.5 ± 3.0
Fasting serum insulin (ng/ml)	1.18 ± 0.22	1.08 ± 0.19	1.01 + 0.13*	1.01 ± 0.15*
HOMA-IR	7.4 ± 0.11	6.7 ± 0.10	6.2 ± 0.08*	6.2 ± 0.11*
Serum TNF-α (pg/mL)	77.8 ± 6.4	62.9 ± 6.2*^†^	58.9 ± 5.4*	54.7 ± 5.1*
Serum AST (IU/L)	44.0 ± 2.1	30.7 ± 2.4*^†^	30.0 ± 2.6*^†^	36.8 ± 2.2*
Serum ALT (IU/L)	42.6 ± 6.3	29.4 ± 8.6*	24.2 ± 4.0*	29.0 ± 5.3*

Amyloid-β_(25–35)_ infused rats were fed a high-fat diet containing dextrin (the AD-CON group), water extract of *Cassiae Semen* (the AD-CS group) or water extract of *Forsythiae Fructus* (the AD-FF group) for 49 days. Amyloid-β_(35–25)_ infused rats were fed a high-fat diet containing dextrin (the CON group). HOMA-IR, Homeostatic model assessment for insulin resistance; TNF-α, tumour necrosis factor-α; AST, aspartate aminotransferase; ALT, alanine aminotransferase. Values represent means ± standard deviations (*n* = 10).

*Significantly different from the AD-CON by Tukey test at *p* < 0.05.

^†^Significantly different from the CON by Tukey test at *p* < 0.05.

**Figure 2.**
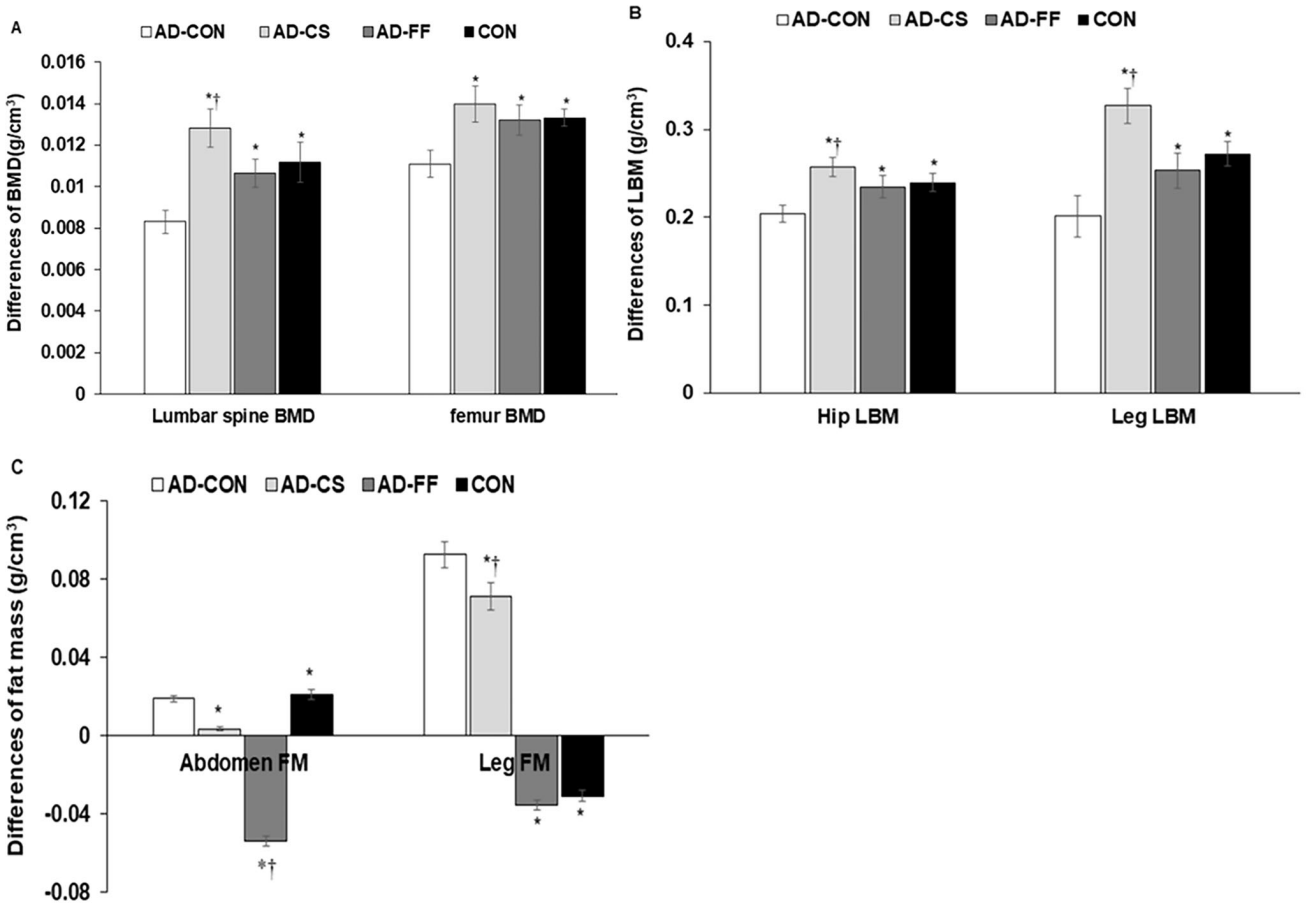
Differences in bone mineral density (BMD), lean body mass (LBM), and fat mass (FM) before hippocampal amyloid-β infusion and at the end of the intervention. Amyloid-β_(25–35)_ infused rats were fed a high-fat diet containing dextrin (AD-CON group), the water extract of *Cassiae Semen* (AD-CS group), or the water extract of *Forsythiae Fructus* (AD-FF) for 49 days. Amyloid-β_(35–25)_ infused rats were fed a high-fat diet containing dextrin (CON group). Before hippocampal amyloid-β infusion and at the end of the intervention, body composition was measured by DEXA, and the differences of BMD, LMD, and FM were calculated. (A) Differences between before and after treatments in the lumbar spine and femur BMD. (B) Differences between before and after treatment in hip and leg LBM. (C) Differences between before and after treatment in abdominal and leg FM. Bars and error bars represent the means ± standard deviations (n = 10). *Significantly different from the AD-CON by Tukey test at *p* < 0.05. ^†^Significantly different from the CON by Tukey test at *p* < 0.05.

BMD loss in the lumbar spine and femur between 1 and 7 weeks of treatments was much higher in the AD-CON group than in the CON group. FF and CS protected against the BMD losses, and BMD was even higher in the AD-CS group than in the CON group (*p* < 0.05; Figure 2A). In the AD-CON group, BMD loss was more significant in the lumbar spine than in the femur, and the protective effect of CS on BMD loss was also higher in the lumbar spine than the femur (*p* < 0.05; Figure 2A). Moreover, hip and leg LBM loss were higher in the AD-CON group than in the CON group, and interestingly, LBM loss was lower in the AD-CS group than in the CON group (Figure 2B). Furthermore, CS supplementation had a more significant impact on leg LBM loss than on hip LBM. However, the abdominal fat mass did not differ between the AD-CON and CON groups, but leg fat mass was higher in the AD-CON than the CON (*p* < 0.05; Figure 2C). AD-CS and AD-FF lowered the abdominal fat mass to less than AD-CON. Fat mass differences in the leg were much lower in the CON than in the AD-CON, whereas AD-CS and AD-FF protected against decreased leg fat mass, compared to the AD-CON (Figure 2C). AD-FF lowered the abdominal and leg fat masses more than AD-CS (Figure 2C).

### Glucose metabolism

Memory impairment is related to brain insulin resistance, which is associated with systemic and brain glucose metabolism, and thus, we investigated glucose metabolism. At the end of the treatment period, serum glucose concentrations in the fasting state were not significant among the four groups (*p* < 0.05; Table 2). However, serum insulin concentrations were higher in the AD-CON group than in the CON group, and FF supplementation reduced serum insulin concentrations in the fasting state to the CON group level (Table 2). HOMA-IR, an insulin resistance index, was higher in the AD-CON group than in the CON group, and FF supplementation blocked the HOMA-IR increases, maintaining HOMA-IR at the CON group level (*p* < 0.05; Table 2). After treatment, serum TNF-α concentration, an index of inflammation, was much higher in the AD-CON group than in the CON group, and CS or FF supplementation decreased TNF-α expression relative to AD-CON (*p* < 0.01; Table 2). FF intake resulted in serum TNF-α concentrations similar to the CON group. AD-Con exhibited high serum AST and ALT concentrations due to increased systemic insulin resistance, and FF and CS reduced them to less than the CON (Table 2). AD increased serum AST and ALT concentrations, but the increment was minimal. FF and CS decreased them to less than the CON group.

**Figure 3.**
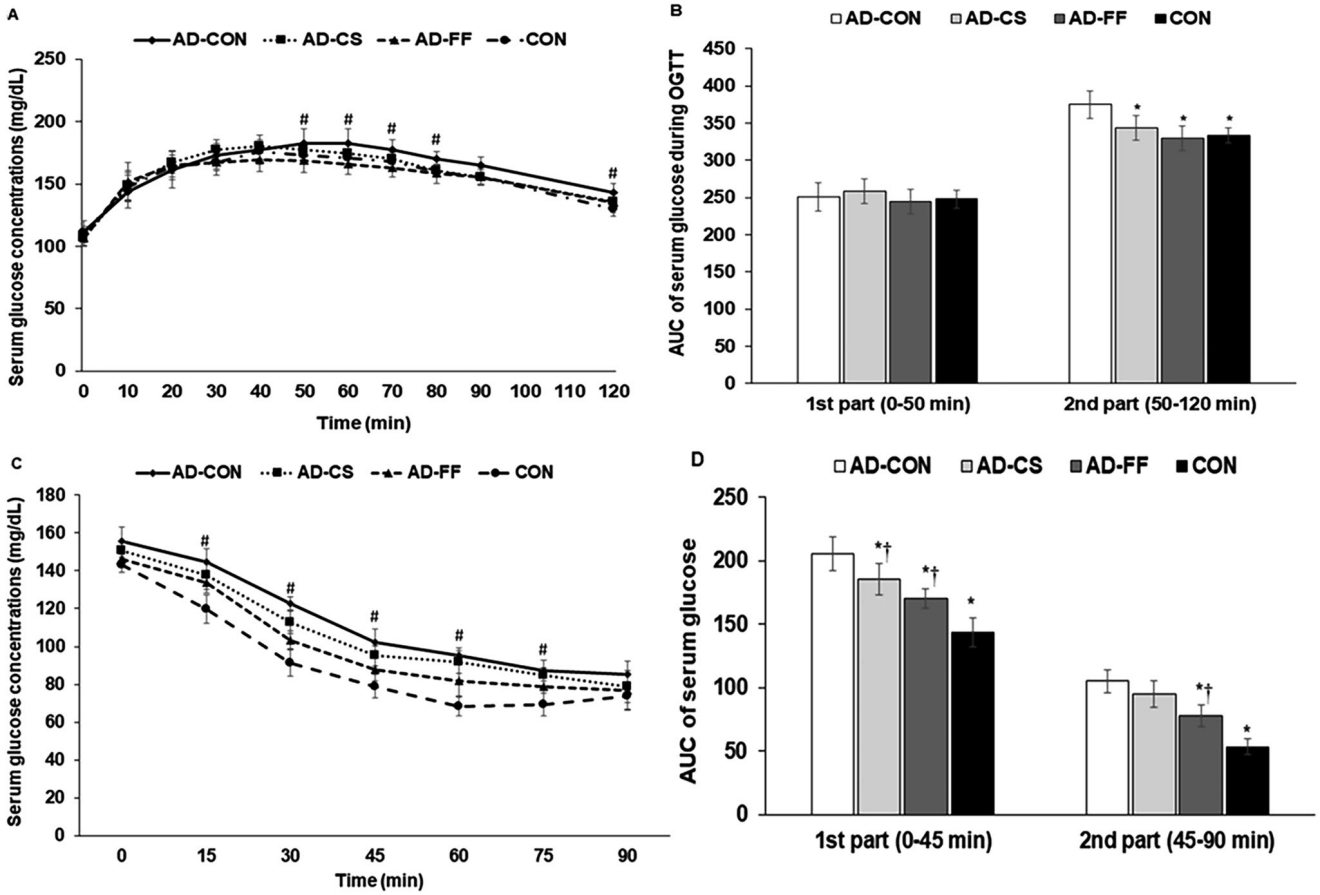
Changes in serum glucose and insulin concentrations and areas under the curve during oral glucose tolerance testing (OGTT) and intraperitoneal insulin tolerance testing (IPITT). Amyloid-β_(25–35)_ infused rats were fed a high-fat diet containing dextrin (AD-CON group), the water extract of *Cassiae Semen* (AD-CS group), or the water extract of *Forsythiae Fructus* (AD-FF) for 49 days. Amyloid-β_(35–25)_ infused rats were fed a high-fat diet containing dextrin (CON group). (A) Changes in serum glucose concentration measured after oral consumption of 2 g glucose/kg body weight (OGTT) after overnight fasting at the fifth week. (B) The area under the curve (AUC) of serum glucose concentration changes during the 1^st^ (0–50 min) and second parts (50–120 min) of OGTT. (C) Changes in serum glucose concentration measured after intraperitoneal injection of 1 IU insulin/kg body weight (IPITT) after 6 h food deprivation at 3 days after OGTT. (D) The area under the curve of serum glucose concentration changes during the 1^st^ (0–30 min) and second parts (30–90 min) of IPITT. Bars and error bars represent the means ± standard deviations (*n* = 10). ^#^ Significantly different among the groups by one-way ANOVA at *p* < 0.05. *Significantly different from the AD-CON by Tukey test at *p* < 0.05. ^†^Significantly different from the CON by Tukey test at *p* < 0.05.

During OGTT, serum glucose concentrations increased for up to 50–60 min and then decreased in all groups (Figure 3A). During the first 40 min, serum glucose concentrations were not significantly different among the four groups, but after 40 min, serum glucose concentrations were higher in the AD-CON group than in the AD-FF and CON groups (*p* < 0.05; Figure 3A). During the 1^st^ part (0–50 min) of OGTT, AUCs were not significantly different among the groups, but during the 2^nd^ part (50–120 min), AUC was higher in the AD-CON than in the CON group (*p* < 0.05). FF or CS supplementation reduced the AUC in the 2^nd^ part to the CON group level (Figure 3B).

After a 6 h fast, serum glucose concentrations were higher in the AD-CON group than in the CON group. After an intraperitoneal insulin injection, serum glucose concentrations were sharply reduced for up to 45 min and then decreased slightly or maintained in all groups (Figure 3C). During IPITT, serum glucose concentrations were lower in the CON group than in the AD-CON group, and FF supplementation lowered serum glucose to below the level in the CON group (*p* < 0.05; Figure 3D). AUC in the 1^st^ part (0–45 min) of the IPITT decreased in the order of AD-CON > AD-CS = AD-FF > CON, and in the 2^nd^ part (45–90 min) decreased in the order of AD-CON = AD-CS > AD-FF > CON (*p* < 0.05). IPITT results revealed systemic insulin resistance, indicating that animals in the AD-CON group had impaired systemic insulin sensitivity compared to those in the CON, AD-CS, and AD-FF groups. Furthermore, FF water extract supplementation reduced insulin resistance more than the CS water extract.

### Amyloid-β deposition in the hippocampus and memory deficits

**Figure 4.**
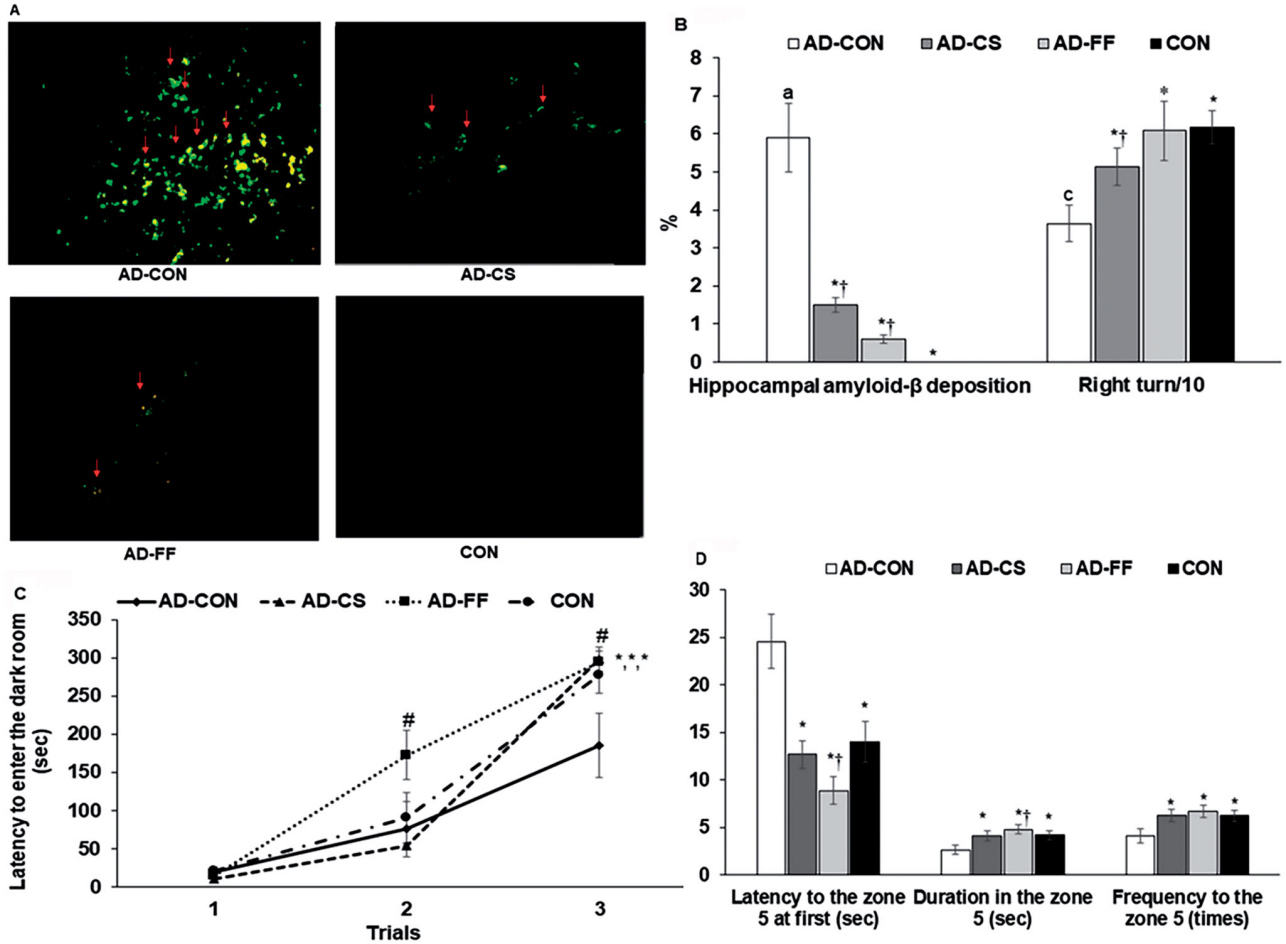
Memory deficits in rats after amyloid-β infusion. The amyloid-β_(25–35)_ infused rats were fed high-fat diets containing dextrin (AD-CON group), water extract of *Cassiae Semen* (AD-CS group), or water extract of *Forsythiae Fructus* (the AD-FF group) for 49 days. The amyloid-β_(35–25)_ infused rats were fed a high-fat diet containing dextrin (CON group). At the end of the experiment, (A) Amyloid-β deposition in the hippocampus by immunohistochemistry method. (B) The percentage of correct alternations to total rotations in the Y maze test on an experimental day 37 from the amyloid-β infusion. (C) Latency time to enter the darkened room in the passive avoidance test 3 times every 8 h on day 41 from the amyloid-β infusion. (D) The frequencies of visiting the zone where the platform was located, time spent in the zone, and time spent finding the target zone in the third trial on day 43 from the amyloid-β infusion. Dots and error bars represent the means ± standard deviations (*n* = 4 for brain amyloid-β deposition; *n* = 10 for memory deficit). *Significantly different from the AD-CON by Tukey test at *p* < 0.05. ^†^Significantly different from the CON by Tukey test at *p* < 0.05.

No amyloid-β deposition was observed in hippocampi in the CON group, and FF and CS supplementation protected against its accumulation compared with the AD-CON group (Figure 4A; *p* < 0.05). The less amyloid-β deposition was observed in the AD-FF group than in the AD-CS group (Figure 4B; *p* < 0.05).

Short-term memory impairment was assessed with passive avoidance and Y maze testing, while spatial memory deficits were assessed by water maze testing (Figure 4B–D). During Y maze tests, the percentage of correct alternations was lower in the AD-CON group than in the CON group. FF and CS supplementation increased correct alternation percentages compared with the AD-CON group, and AD-FF increased the percentages of correct alteration up to the CON (*p* < 0.05; Figure 4B). In the passive avoidance test, rats exhibited similar latencies, but latencies increased after delivering an electric shock, and animals in the AD-FF group had the highest latency in the second trial (*p* < 0.05). In the third trial, latencies were longer in the AD-CS, AD-FF, and CON groups than in the AD-CON group (*p* < 0.05; Figure 4C), indicating that the AD-CS and AD-FF groups improved short-term memory impairment similar to the CON group.

In the water maze test, rats were required to find the platform in zone 5 on two consecutive days, and on the fifth day, the platform was removed (*p* < 0.05; Figure 4D). The latencies to find zone 5 were longer in the AD-CON than CON, and AD-CS and AD-FF showed much shorter latencies than AD-CON, and the latencies were even shorter in AD-FF than CON (Figure 4D). The durations to stay in zone 5 to find the platform were shorter in the AD-CON group than in the CON group on the third trial (*p* < 0.05). Zone 5 visiting frequencies were lower in the AD-CON group than in the CON, and AD-CS and AD-FF increased the frequencies to visit zone 5 up to those of the CON group during the third trial (Figure 4D).

### Hippocampal insulin resistance and neuroinflammation

**Figure 5.**
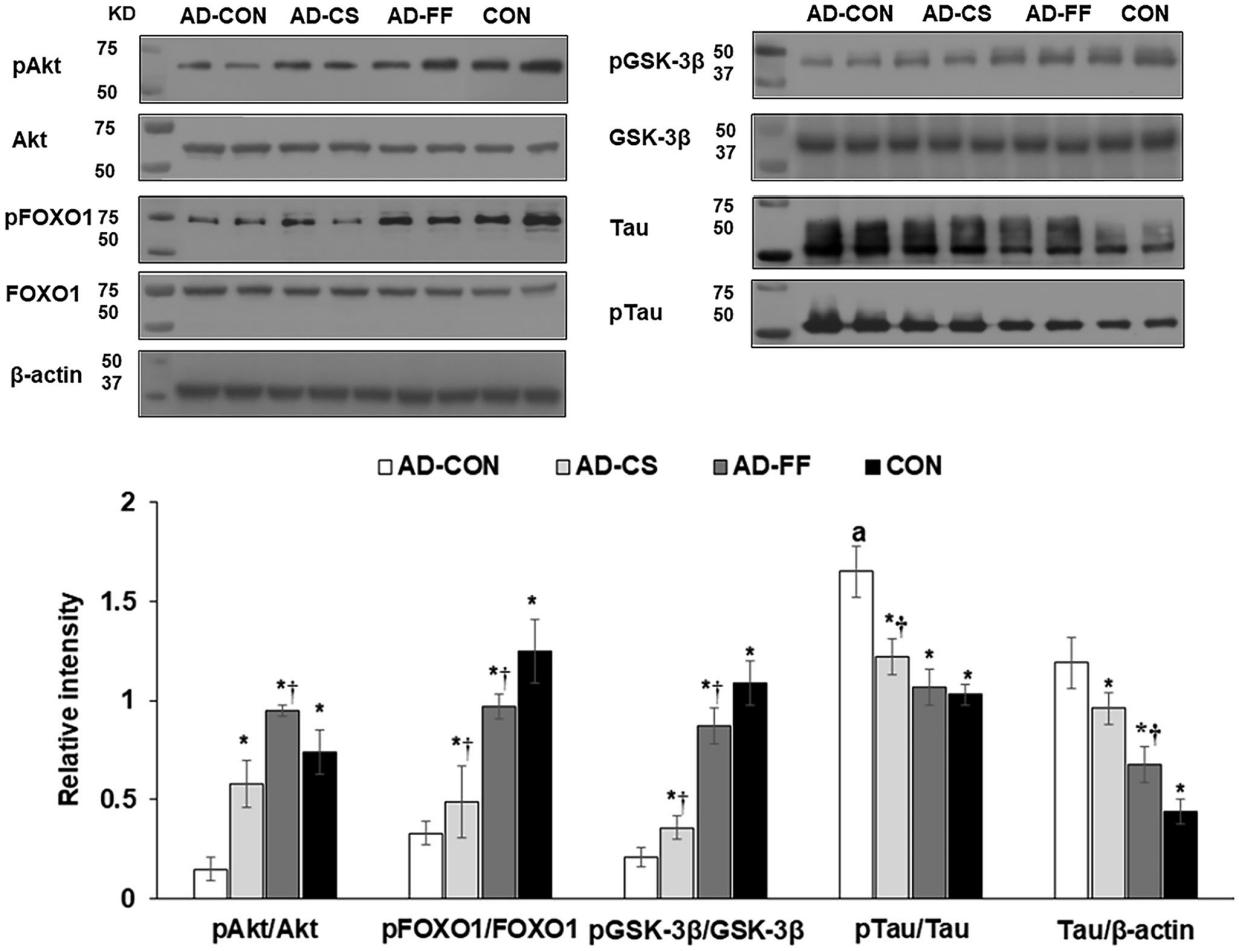
Amyloid-β deposition, expression of brain growth factor, neuroinflammation, and insulin signalling in the hippocampus. Amyloid-β_(25–35)_ infused rats were fed a high-fat diet containing dextrin (AD-CON group), water extract of *Cassiae Semen* (AD-CS), or water extract of *Forsythiae Fructus* (AD-FF group) for 49 days. Amyloid-β_(35–25)_ infused rats were fed a high-fat diet containing dextrin (CON group). Hippocampal insulin signalling (phosphorylation and contents of Akt, GSK-3β, FOXO1, and tau) was measured by immunoblotting. Bars and error bars represent means ± standard deviations (*n* = 4). *Significantly different from the AD-CON by Tukey test at *p* < 0.05. ^†^Significantly different from the CON by Tukey test at *p* < 0.05.

The amyloid-β deposition is associated with brain insulin resistance, oxidative stress, and neuroinflammation, whereas systemic insulin resistance and inflammation are indirectly linked to brain insulin resistance (Daily et al. 2021). Hippocampal triglyceride contents were higher in the AD-Con than the CON, and AD-FF and AD-CS decreased them to as much as the Con (*p* < 0.05; Table 3). By contrast, hippocampal glycogen contents were lower in AD-CON than in the CON, whereas AD-FF and AD-CS were reduced similarly to the CON (Table 3). Hippocampal lipid peroxide contents were much higher in the AD-Con than CON, whereas AD-FF and AD-CS reduced them (Table 3). We found that systemic insulin resistance and inflammation were higher in the AD-CON group than in the CON group. When we investigated hippocampal insulin signalling, the phosphorylation levels of Akt, GSK-3β, and FOXO1 in the hippocampus were lower in the AD-CON group than in the CON group, whereas their protein expressions were not different among all groups (*p* < 0.05; Figure 5). CS or FF supplementation suppressed these reductions in phosphorylation, and FF did so more potently than CS. Tau expression and tau phosphorylation in the hippocampus were much higher in the AD-CON group than in the CON group, and CS or FF supplementation inhibited the increases. However, FF had a more significant effect than CS (Figure 5).

**Table 3.** Liver damage and oxidative stress and neuroinflammation in the hippocampus.

Variables	AD-Con	AD-CS	AD-FF	CON
Hippocampal triglyceride (mg/g)	243 ± 9	209 ± 9*	202 ± 8*	210 ± 8*
Hippocampal glycogen (mg/g liver)	39.3 ± 3.5	55.5 ± 6.8*	54.5 ± 6.5*	54.1 ± 5.9*
Hippocampal lipid peroxides (MDA μmol/g tissue)	0.39 ± 0.05	0.26 ± 0.04*^†^	0.21 ± 0.04*	0.22 ± 0.03*
Relative mRNA expression of hippocampal *TNF-α* (AU)	1.0 ± 0	0.71 ± 0.09*^†^	0.66 ± 0.08*^†^	0.57 ± 0.07*
Relative mRNA expression of hippocampal *IL-1β* (AU)	1.0 ± 0	0.79 ± 0.09*^†^	0.74 ± 0.10*	0.68 ± 0.08*
Relative mRNA expression of hippocampal *BDNF* (AU)	1.0 ± 0	1.6 ± 0.1*	1.8 ± 0.4*	1.7 ± 0.5*
Relative mRNA expression of hippocampal *CNTF* (AU)	1.0 ± 0	2.1 ± 0.5*	2.9 ± 0.4*^†^	2.2 ± 0.7*
Relative mRNA expression of hippocampal *Tau* (AU)	1.0 ± 0	0.76 ± 0.08*^†^	0.67 ± 0.09*	0.66 ± 0.08*

Amyloid-β_(25–35)_ infused rats were fed a high-fat diet containing dextrin (the AD-CON group), water extract of *Cassiae Semen* (the AD-CS group) or water extract of *Forsythiae Fructus* (the AD-FF group) for 49 days. Amyloid-β_(35–25)_ infused rats were fed a high-fat diet containing dextrin (the CON group). MDA, malondialdehyde. In mRNA expression, the CT values of the AD-CON group were used as the reference group. *TNF-α*, tumour necrosis factor-α; *IL-1β*, interleukin-1β; *BDNF*, brain-derived neurotropic factor; *CNTF*, ciliary neurotrophic factor. Values represent means ± standard deviations (*n* = 6 for triglyceride and glycogen contents; *n* = 3 for mRNA expression).

*Significantly different from the AD-CON by Tukey test at *p* < 0.05.

^†^Significantly different from the CON by Tukey test at *p* < 0.05.

Hippocampal mRNA expression of *TNF-α* and *IL-1β* (markers of neuroinflammation) was higher in the AD-CON group than in the CON, AD-CS, and AD-FF groups. CS and FF water extract supplementation reduced *TNF-α* mRNA expression but not to the CON group level (*p* < 0.01; Table 3). These results show that CS and FF water extracts reduced neuroinflammatory cytokine expression, indicating that they suppressed amyloid-β deposition by promoting hippocampal insulin signalling and attenuating neuroinflammation. Furthermore, *BDNF* and *CNTF* play crucial roles in the survival of neuronal cells associated with memory impairment. *BDNF* mRNA expression was much lower in the AD-CON group than in the CON group, and CS or FF supplementation prevented the reduction (*p* < 0.05; Table 3). The expression pattern of *CNTF* mRNA was similar to that of *BDNF*, and CS supplementation increased *CNTF* mRNA expression to a level higher than that in the CON group (*p* < 0.01; Table 3). *Tau* mRNA expression in the hippocampus was higher in the AD-CON group than in the CON group, and CS or FF water extract supplementation protected against AD-induced increases in the *Tau* mRNA expression (Table 3). Hippocampal *Tau* mRNA expressions were higher in the AD-FF than AD-CS and CON groups (*p* < 0.05; Table 3).

### Gut microbiome

**Figure 6.**
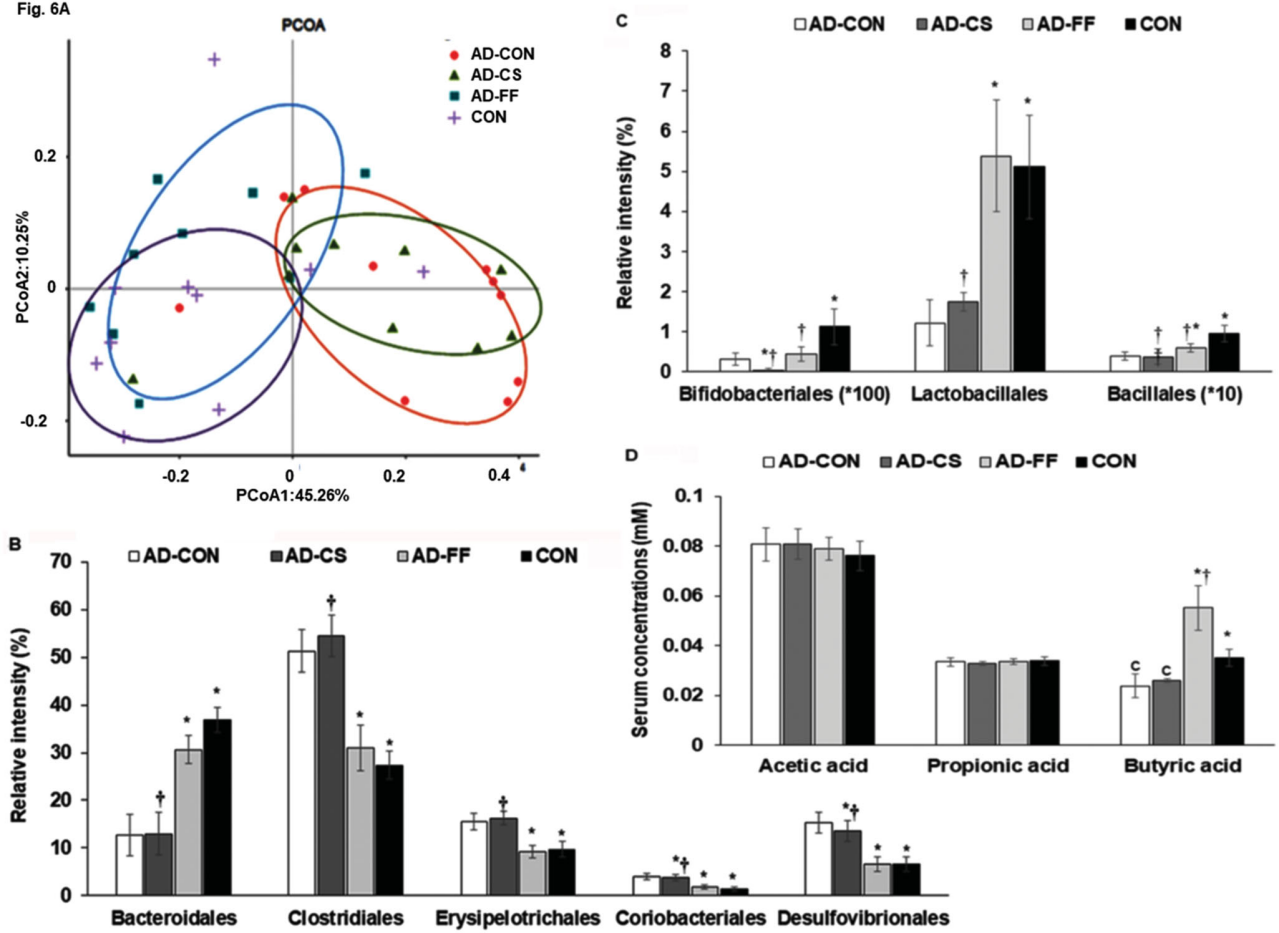
Gut microbiome profiles. Faeces were collected from the caeca at the end of the experiment, and bacterial DNA was analysed using the NGS method. Amyloid-β_(25–35)_ infused rats were fed a high-fat diet containing dextrin (AD-CON group), water extract of *Cassiae Semen* (AD-CS group), or water extract of *Forsythiae Fructus* (AD-FF group) for 49 days. Amyloid-β_(35–25)_ infused rats were fed a high-fat diet containing dextrin (CON group). The microbiome of faeces in the caecum was measured by the NGS method at the end of the study. Short-chain fatty acid (SCFA) concentrations in the serum from the portal vein were measured by gas chromatography. (A) Principal coordinate analysis (PCoA) of faecal bacteria. (B) Relative amounts (%) of *Bacteriodales, Clostridales*, and *Erysipelotrichales* in faecal samples. (C) Relative amounts (%) of *Lactobacillales, Desulfovibrionales,* and *Enterobacteriales* in faecal samples. (D) Serum concentrations of SCFA as determined by gas chromatography. Bars and error bars represent the means ± standard deviations (*n* = 10). *Significantly different from the AD-CON by Tukey test at *p* < 0.05. ^†^Significantly different from the CON by Tukey test at *p* < 0.05.

Principal coordinate analysis (PCoA) showed separated clustering of faecal bacterial communities among the four treatment groups (Figure 6A; *p* < 0.001). Gut bacterial communities in the AD-CON and CON groups were clearly separated, whereas communities in the AD-FF group were separated from the AD-CON group but not the AD-CS group (Figure 6A).

Furthermore, analysis of the molecular variance (AMOVA) showed that the bacterial communities in faeces were significantly different among the four groups (*p* < 0.01). The proportions *Erysipelotrichales*, *Clostridiales*, *Coriobacteriales*, and *Desulfovibrionales*, were higher in the AD-CON group than in the CON and AD-FF groups (Figure 6B), but they were similar in the AD-CS and AD-CON groups. Proportions of *Bacteroidales, Lactobacillales,* and *Bacillales* were lower in the AD-CON group than in the CON group (*p* < 0.05). Furthermore, FF better protected against their decreases in the AD-CON rats (Figure 6C). Serum acetate and propionate concentrations were not significantly different among the groups. However, serum butyrate concentrations were lower in the AD-CON than the CON, whereas they were much higher in AD-FF than AD-CON and CON. Serum butyrate concentrations did not differ between AD-CS and AD-CON, suggesting CS might not change the relative abundance of butyrate-producing bacteria (Figure 6D).

## Discussion

Although its aetiology remains unclear, AD is associated with neuronal cell death in the hippocampus caused by amyloid-β and tau accumulation (Bloom 2014). As yet, no drug has been developed that can slow the progression of AD despite much research on the topic spanning several decades (Hodson 2018). The present study showed that CS and FF could ameliorate memory deficits caused by amyloid-β deposition in the hippocampus, but FF was more effective than CS. The differences between CS and FF for mitigating memory improvement were related to the gut microbiome.

In a preliminary *in vitro* study, both CS and FF at 1–50 μg/mL dose-dependently protected NGF-differentiated PC12 cells from amyloid-β-induced cell death, and at 10–20 μg/mL, both increased the mRNA expressions of *BDNF* and *CNTF* (biomarkers of neuronal cell survival) and decreased tau mRNA expression. Based on cell culture results, their utilisation rates (20%) and half-life (about 4 h), a safe and effective daily dosage were estimated to be 200 mg/kg body weight of CS, FF, and dextrin for the animal study, which was the equivalent of 1–1.5 g per day in humans using a conversion factor between rodents and humans (Committee of FDA 2005; Manach et al. 2005).

Consistent with the previous studies showing that AD is accompanied by progressive weight loss with decreased BMD and LBM mainly in the early stage of AD, but increase the fat mass (Buffa et al. 2014; Ingenbleek and Bernstein 2015; Pu et al. 2020), the present study showed that the BMD and LBM were lower in the AD-CON than the CON in DEXA analysis. AD-CS and AD-FF protected against their decrease compared to the AD-CON, whereas AD-CS was better protected against BMD and LBM losses than AD-FF. However, inconsistent with previous studies (Pu et al. 2020), epididymal fat and retroperitoneal fat mass did not differ between the AD-CON and the CON groups in the present study. In the DEXA analysis, the abdominal fat mass also showed similar results as directly measured visceral fat mass. Alzheimer’s disease insignificantly increased visceral fat mass, but the amounts were small and fell short of showing any differences in the present study. However, the fat mass in the abdomen was lower in AD-CS and AD-FF than AD-CON. Thus, AD was related to the changes in BMD, LBD, and fat mass, and both CS and FF suppressed the modulation of body composition, which may have been involved in protecting against neuronal cell death.

Amyloid-β_(1–42)_ is the full-length amyloid, and amyloid-β_(25–35)_ is the segment critical for amyloid-β aggregation and neuroinflammation. Bilateral hippocampal infusion of amyloid-β_(25–35)_ in rats has been reported to induce learning and memory impairments (Park et al. 2017a; Zeng et al. 2019). In the present study, amyloid-β_(25–35)_ infusion into the hippocampus induced amyloid-β deposition and short-term and spatial memory impairments, whereas amyloid-β_(35–25)_ infusion did not elicit these effects. Amyloid-β_(25–35)_ also increased TNF-α and IL-1β in the hippocampus, suggesting NF-κB activation and neuroinflammation. Interestingly, neuroinflammation is also influenced by the gut microbiome-brain axis through modulating serum SCFA concentrations (Cerovic et al. 2019; Daily et al. 2021). Furthermore, amyloid-β aggregation is associated with tau hyperphosphorylation and is inhibited by the potentiation of insulin signalling through GSK-3β (Laurent et al. 2018; Zhang et al. 2018b), which is a crucial modulator of tau phosphorylation and a downstream protein in the insulin→phosphoinositide 3-kinase→protein kinase B signalling pathway (Zhang et al. 2018b; Gabbouj et al. 2019). In the present study, the hippocampal tissues of AD-CON rats exhibited reduced phosphorylations of Akt, GSK-3β, and FOXO1. AD-CON rats also exhibited increased tau expression and phosphorylation, indicating the presence of tau pathology, which showed amyloid-β_(25–35)_ infusion increased neuroinflammation, attenuated insulin signalling, and induced amyloid-β deposition leading to memory impairment. CS and FF supplementation suppressed the potentiation of neuroinflammation and the attenuation of hippocampal insulin signalling in AD-induced rats. FF had the most significant impact on insulin signalling and inflammation in the hippocampus in the present study.

CS and FF contain polyphenols, flavonoids, and other phytochemicals, which have been reported to alleviate Alzheimer’s disease-like symptoms in animal models (Schimidt et al. 2017; Park et al. 2017a). Herbs such as CS and FF also frequently have antioxidant and anti-inflammatory activities and reduce systemic insulin resistance. However, polyphenols beneficially suppress amyloid-β accumulation in the brain, although they have not been detected in brain tissues. There could be several explanations for this apparent contradiction: bacteria in the gastrointestinal tract metabolise polyphenols into other phenolic entities, and the metabolites are delivered into the brain, and they may affect amyloid-β accumulation through modulating enzymes and receptors. Furthermore, the herbal components can provide functions mediated by the gut microbiota-liver-brain axis (Daily et al. 2021). The present study showed that FF modulated gut microbiota composition to promote the gut microbiome-brain axis, reduced neuroinflammation, and alleviated AD symptoms better than CS.

Herbal medicine has been mainly reported to induce liver toxicity as an adverse effect (Posadzki et al. 2013). CS and FF intake did not show any adverse effects in the present study. However, in the present study, CS and FF prevented the increase in liver enzymes in the AD-Con group, and they reduced serum AST concentration to lower levels than the CON. These results indicated that a high-fat diet and AD caused liver damage, and CS and FF prevented the liver damage. Furthermore, the brain damage may be linked to the liver, and thus CS and FF may have alleviated the brain and liver damage through the brain-liver axis. Previous studies have demonstrated that systemic and brain insulin resistance are connected through the brain-liver axis (Campbell et al. 2018; Boccardi et al. 2019). Notably, accumulating evidence indicates that metformin, which is used to treat type 2 diabetes by improving insulin resistance, may have therapeutic potential for AD (Campbell et al. 2018; Boccardi et al. 2019; Farr et al. 2019).

FF contains various phenylethanoid glycosides, lignans, flavonoids, aliphatic alcohols, iridoids, diterpenoids, triterpenoids, and alkaloids (Dong et al. 2017b). Phenylethanoid glycosides, such as forsythosides, are the major bioactive constituents of FF and have antioxidant, anti-inflammatory, antibacterial, and antiviral activities. Consistent with the present study, FF has shown neuroprotective activities *in vitro* and *in vivo*: forsythoside A, a primary component of FF, significantly inhibits cell apoptosis by downregulating acetylcholinesterase activity in the PC12 and HT22 cells administered with amyloid-β_(25–35)_ (Yan et al. 2017). Administration of 10 mg FF/kg body weight ameliorates cognitive dysfunction caused by global cerebral ischaemia by inhibiting the activations of microglia and astrocytes in gerbils (Kim et al. 2011). In previous metabolomics studies, FF intake has not increased the primary components of FF, including oleanolic acid, betulinic acid, ursolic acid, oleanolic acid-3-acetate, arctiin, and isorengyol in the circulation (Bao et al. 2016). However, FF intake has elevated formylan-thranilic acid, nicotinic acid mononucleotide, lysophosphatidylcholine, and phosphatidylcholine in the circulation, which was the metabolites of FF components (Jia et al. 2015; Bao et al. 2016). Thus, the changes of FF metabolites may improve memory deficit in an AD animal model.

CS primarily contains anthraquinones (emodin, chrysophanol, physcion, aloe-emodin, rhein, and obtusina), naphthopyrones (torachrysone, rubrofusarin, and rubrofusarin-6-*O*-β-d-gentiobioside, etc.), and volatile oils (*E*-9-octadecenoic acid, *n*-hexadecanoic acid, 9,10-anthracenedione, and 1,8-dihydroxy-3-methyl). Unlike FF, CS intake has been reported to increase plasma concentrations of anthraquinone aglycones, aurantio-obtusin, obtusifolin, questin, 2-hydroxyemodin-1-methyl-ether, and rhein (Guo et al. 2017). Furthermore, CS and its components have been demonstrated to possess antidiabetic, hepatoprotective, and neuroprotective effects by reducing oxidative stress and inflammation (Zhang et al. 2018a; Meng et al. 2019; Wang et al. 2019). However, the effects of FF and CS or their components on amyloid-β deposition in amyloid-β infused animals have not been previously investigated. The present study shows that both FF and CS decreased amyloid-β deposition by reducing amyloid-β-induced insulin signalling attenuation and neuroinflammation. In addition, FF better protected against memory impairment than CS by reducing amyloid-β deposition more than CS and did so in association with potentiating hippocampal insulin signalling and reducing neuroinflammation.

Intestinal microbiome dysbiosis elevates systemic inflammation and dysregulates the gut-brain axis, which, in turn, exacerbates neuroinflammation and leads to dementia and AD through the gut microbiota-brain axis. Initially, intestinal microbiome dysbiosis induces local and systemic inflammation and dysregulation of the gut-brain axis. Subsequently, increased permeability of the gut epithelial barrier results in the invasion of different bacteria, viruses, and their neuroactive products that support neuroinflammatory reactions in the brain (Karki et al. 2017; Farr et al. 2019). SCFAs are modulators of the gut-brain axis. Serum acetate concentrations were reported to be an index of insulin resistance, and serum propionate and butyrate concentrations are reliable indicators of increases in beneficial bacteria in the gut (Fu et al. 2019). Butyrate is the primary energy source for colonocytes and directly influences colonocyte differentiation. It also contributes to enhancing intestinal barrier function and mucosal immunity (Fu et al. 2019). Thus, it is crucial to increase butyrate production by the intestinal gut microbiome. In the present study, the gut microbiome was separated between the AD-CON and CON groups, gut microbiomes in the AD-CS and AD-CON groups were not separated, and the gut microbiomes in the AD-FF and CON groups were similar. Furthermore, serum butyric acid concentrations differed in the AD-CON and CON groups, whereas serum acetate and propionate concentrations were not significantly different among the four treatment groups. Serum butyrate concentrations were higher in the AD-FF group than in the AD-CON and CON groups.

## Conclusions

Dietary supplementation with FF or CS protected against memory impairment in an AD rat induced by amyloid-β infusion into the hippocampus. This protective effect was associated with the potentiation of hippocampal insulin signalling and reduced oxidative stress and neuroinflammation. Furthermore, FF, but not CS, improved the gut microbiome community and increased serum butyrate concentrations, suggesting that FF promoted the gut-brain axis, which improved memory function in an AD rat model. FF improved memory functions better than CS by enhancing the gut-brain axis. Future research should be conducted to identify FF active compounds to make a standardised extract. There should also be clinical research in humans to establish safety, efficacy, and dose dependence in human subjects. This research establishes the potential for neuroprotective benefits from FF against AD in a rat model and justifies additional research in humans.
